# Combining the receptor tyrosine kinase inhibitor AEE788 and the mammalian target of rapamycin (mTOR) inhibitor RAD001 strongly inhibits adhesion and growth of renal cell carcinoma cells

**DOI:** 10.1186/1471-2407-9-161

**Published:** 2009-05-27

**Authors:** Eva Juengel, Johanna Engler, Iyad Natsheh, Jon Jones, Ausra Mickuckyte, Lukasz Hudak, Dietger Jonas, Roman A Blaheta

**Affiliations:** 1 Klinik für Urologie und Kinderurologie, Zentrum der Chirurgie, Johann Wolfgang Goethe-Universität, Frankfurt am Main, Germany

## Abstract

**Background:**

Treatment options for metastatic renal cell carcinoma (RCC) are limited due to resistance to chemo- and radiotherapy. The development of small-molecule multikinase inhibitors has now opened novel treatment options. We evaluated the influence of the receptor tyrosine kinase inhibitor AEE788, applied alone or combined with the mammalian target of rapamycin (mTOR) inhibitor RAD001, on RCC cell adhesion and proliferation *in vitro*.

**Methods:**

RCC cell lines Caki-1, KTC-26 or A498 were treated with various concentrations of RAD001 or AEE788 and tumor cell proliferation, tumor cell adhesion to vascular endothelial cells or to immobilized extracellular matrix proteins (laminin, collagen, fibronectin) evaluated. The anti-tumoral potential of RAD001 combined with AEE788 was also investigated. Both, asynchronous and synchronized cell cultures were used to subsequently analyze drug induced cell cycle manipulation. Analysis of cell cycle regulating proteins was done by western blotting.

**Results:**

RAD001 or AEE788 reduced adhesion of RCC cell lines to vascular endothelium and diminished RCC cell binding to immobilized laminin or collagen. Both drugs blocked RCC cell growth, impaired cell cycle progression and altered the expression level of the cell cycle regulating proteins cdk2, cdk4, cyclin D1, cyclin E and p27. The combination of AEE788 and RAD001 resulted in more pronounced RCC growth inhibition, greater rates of G0/G1 cells and lower rates of S-phase cells than either agent alone. Cell cycle proteins were much more strongly altered when both drugs were used in combination than with single drug application. The synergistic effects were observed in an asynchronous cell culture model, but were more pronounced in synchronous RCC cell cultures.

**Conclusion:**

Potent anti-tumoral activitites of the multikinase inhibitors AEE788 or RAD001 have been demonstrated. Most importantly, the simultaneous use of both AEE788 and RAD001 offered a distinct combinatorial benefit and thus may provide a therapeutic advantage over either agent employed as a monotherapy for RCC treatment.

## Background

Renal cell carcinoma (RCC) has an extremely poor prognosis with a third of patients presenting with metastatic disease at primary diagnosis and approximately 40% experiencing tumor recurrence after surgical treatment for localized disease. Treatment regimens for metastatic disease included surgical tumor size reduction, followed by immunotherapy. However, the response rate in patients with immunological approaches remains below 10 to 15% and life is prolonged only in highly selected patients [1].

During recent years small-molecule multikinase inhibitors have been developed which target ligands at the molecular level and which may provide a disease-specific therapy for patients with advanced forms of RCC. Indeed, a profound improvement was seen in a trial comparing sunitinib that inhibits the vascular endothelial growth factor (VEGF) receptor and related receptors with interferon-alpha (IFNa) in previously untreated patients with RCC [2].

However, although a higher objective response rate was seen in the sunitinib arm, as was a longer progression-free survival time, 13% of the patients died in the sunitinib arm versus 17% in the IFNa arm which was not significant in this analysis (it should be noted that crossover to the sunitinib arm was allowed, which may mask any ultimate survival benefit). Similarly, sorafenib, another VEGF receptor tyrosine kinase inhibitor, given as second line treatment in a placebo-controlled trial, caused a response in 10% of patients but the difference in survival was not statistically significant [3].

There is also biologic rationale for targeting the epidermal growth factor (EGF) receptor for the treatment of RCC. Still, clinical trials to date have yielded disappointing results. Lapatinib prolonged overall survival and showed a trend towards improved time to progression in a subgroup of patients with tumors that overexpressed the EGF receptor (compared to standard hormone therapy) [4]. Gefitinib (Iressa) did not induce objective responses in a small cohort of relapsed RCC but disease control was observed in 53.8% of patients [5].

Obviously, the present concept of targeted therapy provides delayed progression and extended survival, however, responses are mostly partial and of limited duration. Since aberrant cancer-causing pathways address multiple components, we assume that single drug treatment may not be sufficient for long-term control of RCC, either due to the development of resistance or due to the development of compensatory feedback loops. In fact, it has recently been observed that blockade of the EGF receptor signaling was compensated by an enhanced VEGF synthesis, providing an important survival advantage of VEGF receptor expressing tumor cells [6,7].

The cross-communication between EGF and VEGF signaling suggests that associated targeting of both receptor types may be an adequate approach to block RCC growth and progression. Surprisingly, combined anti-EGF and anti-VEGF receptor agents seem not be sufficient to achieve a distinct therapeutic benefit in cancer patients [8]. Thus, additional intra-tumoral events correlated to RCC progression should be considered when designing a powerful treatment strategy.

Novel data have shown that RCC exhibits constitutive activation of the phosphatidylinositol 3-kinase (PI3K) – Akt – mammalian target of rapamycin (mTOR) pathway, the downstream effector of VEGF and EGF receptor signaling [9,10]. Most importantly, the PI3K-Akt-mTOR pathway is an important mediator of resistance to conventional chemotherapy and to targeted therapy based on EGF or VEGF receptor tyrosine kinase inhibitors [11].

We concluded from these reports that both horizontal and vertical down-regulation of growth factor receptor related signaling may be required to optimize the current protocol of tumor targeting. Particularly, simultaneous blocking of EGF and VEGF receptor activation combined with Akt-mTOR inhibition may profoundly increase the magnitude and duration of anti-tumor effects exerted by single agent application. To proof this hypothesis, we evaluated the influence of the orally available mTOR inhibitor RAD001 (everolimus), applied alone or combined with the dual EGF and VEGF receptor tyrosine kinase inhibitor AEE788 [12], on RCC cell adhesion and proliferation in vitro. Our results indicate that both AEE788 and RAD001 exert potent anti-tumor activity. However, combined use of both compounds seems to be more effective than the single drug application and thus may provide a therapeutic advantage over either agent as monotherapy for RCC treatment.

## Methods

### Cell cultures

Kidney carcinoma Caki-1 and KTC-26 cells were purchased from LGC Promochem (Wesel, Germany). A498 cells were derived from CLS (Heidelberg, Germany). Tumor cells were grown and subcultured in RPMI 1640 medium (Seromed, Berlin, Germany) supplemented with 10% FCS, 100 IU/ml penicillin and 100 μg/ml streptomycin at 37°C in a humidified, 5% CO_2 _incubator. Endothelial cells (HUVEC) were isolated from human umbilical veins and harvested by enzymatic treatment with chymotrypsin. HUVEC were grown in Medium 199 (Biozol, Munich, Germany), 10% fetal calf serum (FCS; Gibco, Karlsruhe, Germany), 10% pooled human serum (Blood Bank of The German Red Cross, Frankfurt am Main, Germany), 20 μg/ml endothelial cell growth factor (Boehringer, Mannheim, Germany), 0.1% heparin (Roche, Basel, Switzerland), 100 ng/ml gentamycin (Gibco) and 20 mM HEPES-buffer (Seromed, Berlin, Germany). Cell cultures were serially passaged. Subcultures from passages 2–4 were selected for experimental use.

### Drugs

AEE788 and RAD001 (provided by Novartis Pharma AG, Basel, Switzerland) were dissolved in DMSO as 10 mM stocks and stored as aliquots at -20°C. RCC cells were treated either with AEE788 or with RAD001 at concentrations indicated in the results section. Combination treatment with both compounds was based on 1 μM AEE788 and 1 nM RAD001. Controls remained untreated. In additional experiments, AEE788 was compared to tyrosine kinase inhibitors which are currently in clinical use: gefitinib, erlotinib or sunitinib (LC Laboratories, Woburn, MA, USA; 1 μM each). To exclude toxic effects of the compounds, cell viability was determined by trypan blue (Gibco/Invitrogen). For apoptosis detection the expression of Annexin V/propidium iodide (PI) was evaluated using the Annexin V-FITC Apoptosis Detection kit (BD Pharmingen, Heidelberg, Germany). Tumor cells were washed twice with PBS, and were then incubated with 5 μl of Annexin V-FITC and 5 μl of PI in the dark for 15 min at RT. Cells were analyzed on a FACScalibur (BD Biosciences, Heidelberg, Germany). The percentage of apoptotic cells (early and late) in each quadrant was calculated using CellQuest software (BD Biosciences).

### Tumor cell adhesion

To analyze tumor cell adhesion, HUVEC were transferred to 6-well multiplates (Falcon Primaria; BD Biosciences) in complete HUVEC-medium. When confluency was reached, Caki-1, KTC-26 or A498 cells were detached from the culture flasks by accutase treatment (PAA Laboratories, Cölbe, Germany) and 0.5 × 10^6 ^cells were then added to the HUVEC monolayer for 60 min. Subsequently, non-adherent tumor cells were washed off using warmed (37°C) Medium 199. The remaining cells were fixed with 1% glutaraldehyde. Adherent tumor cells were counted in five different fields of a defined size (5 × 0.25 mm^2^) using a phase contrast microscope and the mean cellular adhesion rate was calculated.

### Attachment to extracellular matrix components

6-well plates were coated with collagen G (extracted from calfskin, consisting of 90% collagen type I and 10% collagen type III; Seromed; diluted to 100 μg/ml in PBS), laminin (derived from the Engelbreth-Holm-Swarm mouse tumor; BD Biosciences; diluted to 50 μg/ml in PBS), or fibronectin (derived from human plasma; BD Biosciences; diluted to 50 μg/ml in PBS) overnight. Unspecific cell binding was evaluated by culture plates treated with Poly-D-Lysin (Nunc, Wiesbaden, Germany). Plastic dishes served as the background control. Plates were washed with 1% BSA (bovine serum albumin) in PBS to block nonspecific cell adhesion. Thereafter, 0.5 × 10^6 ^tumor cells were added to each well for 60 min. Subsequently, non-adherent tumor cells were washed off, the remaining adherent cells were fixed with 1% glutaraldehyde and counted microscopically. The mean cellular adhesion rate, defined by adherent cells_coatedwell _– adherent cells_background_, was calculated from five different observation fields.

### Measurement of tumor cell growth

Cell proliferation was assessed using the 3-(4,5-dimethylthiazol-2-yl)-2,5-diphenyltetrazolium bromide (MTT) dye reduction assay (Roche Diagnostics, Penzberg, Germany). Treated versus non-treated Caki-1, KTC-26 or A498 cells (100 μl, 1 × 10^4 ^cells/ml) were seeded onto 96-well tissue culture plates. After 24, 48 and 72 h, MTT (0.5 mg/ml) was added for an additional 4 h. Thereafter, cells were lysed in a buffer containing 10% SDS in 0.01 M HCl. The plates were allowed to stand overnight at 37°C, 5% CO_2_. Absorbance at 570 nm was determined for each well using a microplate ELISA reader. Each experiment was done in triplicate. After subtracting background absorbance, results were expressed as mean cell number.

### Cell cycle analysis

Caki-1 or A498 cells were grown to 70% confluency and then treated with AEE788 or with RAD001 or with both AEE788 + RAD001 (controls remained untreated). Cell cycle analyses were carried out after 24 h using both asynchronous and synchronous cell populations. Caki-1 or A498 cells were synchronized at the G1-S boundary with aphidicolin (1 μg/ml) 24 h before starting cell cycle analysis and subsequently resuspended in fresh (aphidicolin free) medium for 2 h. Asynchronous or synchronous tumor cell populations were stained with propidium iodide using a Cycle TEST PLUS DNA Reagent Kit (Becton Dickinson) and then subjected to flow cytometry with a FACScan flow cytometer (Becton Dickinson). 10,000 events were collected from each sample. Data acquisition was carried out using Cell-Quest software and cell cycle distribution calculated using the ModFit software (Becton Dickinson). The number of gated cells in G1, G2/M or S-phase was presented as %.

### Western Blot Analysis

Cell cycle regulating proteins were explored in asynchronous and synchronous tumor cell populations. Tumor cell lysates were applied to a 7% polyacrylamide gel and electrophoresed for 90 min at 100 V. The protein was then transferred to nitrocellulose membranes. After blocking with non-fat dry milk for 1 h, the membranes were incubated overnight with the following monoclonal antibodies: Cdk2 (IgG2a, clone 55, dilution 1:2.500), cdk4 (IgG1, clone 97, dilution 1:250), cyclin D1 (IgG1, clone G124–326, dilution 1:250), cyclin E (IgG1, clone HE12, dilution 1:200), p27 (IgG1, clone 57, dilution 1:500; all: BD Biosciences, Heidelberg, Germany). HRP-conjugated goat-anti-mouse IgG (Upstate Biotechnology, Lake Placid, NY, USA; dilution 1:5.000) served as the secondary antibody. The membranes were briefly incubated with ECL detection reagent (ECL™, Amersham/GE Healthcare, München, Germany) to visualize the proteins and exposed to an x-ray-film (Hyperfilm™ EC™, Amersham/GE Healthcare). β-actin (1:1.000; Sigma, Taufenkirchen, Germany) served as the internal control.

For control purposes, EGF receptor and mTOR signaling were evaluated. A498 or Caki-1 cells were treated with AEE788 or RAD001 or with the AEE788-RAD001 combination for 24 h. Cells were then kept for 2 h in serum-free cell culture medium and subsequently stimulated for 30 min with EGF (100 ng/ml). The following monoclonal antibodies were used: Akt (IgG1, clone 55, dilution 1:500), phospho Akt (IgG1, clone 104A282, dilution 1:500), ERK1 (IgG1, clone MK12, dilution 1:5000), ERK2 (IgG2b, clone 33, dilution 1:5000), phospho ERK1/2 (IgG1, clone 20A, dilution 1:1000), EGFr (IgG1, clone 13/EGFR, dilution 1:500), phospho EGFr (IgG1, clone 74, dilution 1:1000; all: BD Biosciences), p70S6K (IgG, clone 49D7, dilution 1:1000), phospho p70S6K (IgG, clone 108D2, dilution 1:1000; all: New England Biolabs, Frankfurt, Germany).

### Statistics

All experiments were performed 3–6 times. Statistical significance was investigated by the Wilcoxon-Mann-Whitney-U-test. Differences were considered statistically significant at a p value less than 0.05.

## Results

### Dose-response analysis

**Figure 1:**
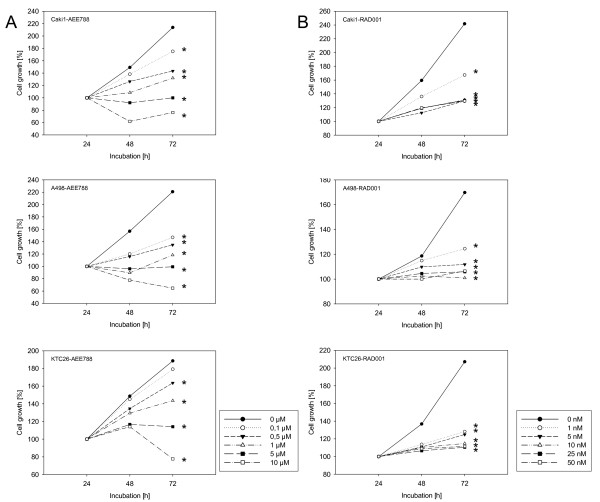
**Effects of AEE788 (1a) or RAD001 (1b) on kidney cancer proliferation *in vitro***. A498, Caki-1 or KTC-26 cells were treated with various concentrations of AEE788 or RAD001, or remained untreated (control). Cell proliferation was then assessed using the MTT dye reduction assay. Cell numbers at day 2 and 3 (48 h and 72 h) were compared to the number on day 1 (24 h, as 100%). One representative of 6 experiments is shown. * indicates significant difference to controls (p < 0.05). SD_intraassay _< 15%.

AEE788 or RAD001 were added to RCC cell cultures and proliferation quantified 24, 48 and 72 h after plating. To clearly interpret and compare cellular growth characteristics, 24 h counts were all set at 100%. Incubation with AEE788 dose-dependently and significantly down-regulated RCC cell proliferation (fig. 1a). 5 μM AEE788 completely stopped RCC cell growth. Based on these data, the sub-optimal concentration of 1 μM AEE788 was chosen for subsequent combination experiments. Fig. 1b demonstrates the influence of RAD001 on RCC growth characteristics. Maximum effects were induced when cells were exposed to 5 nM (A498, Caki-1) or 10 nM RAD001 (KTC-26). The trypan blue assay revealed no signs of drug toxicity. For ongoing studies, the sub-optimal concentration of 1 nM RAD001 was used.

### RCC adhesion to HUVEC or immobilized extracellular matrix proteins

**Figure 2:**
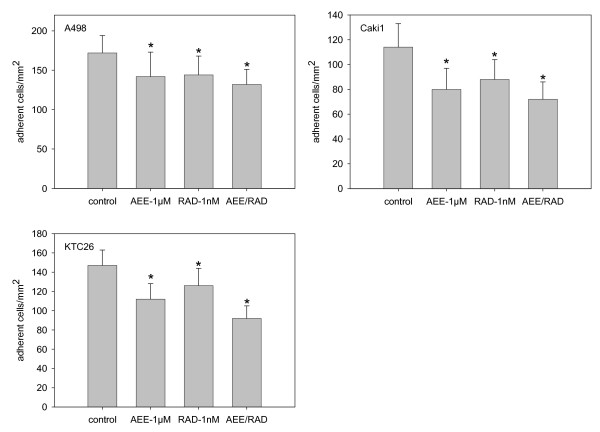
**Adhesion of RCC cells to HUVEC**. A498, Caki-1 or KTC-26 cells were treated with 1 μM AEE788 or 1 nM RAD001, applied alone or in combination. After a 1 h pre-incubation, tumor cells were added at a density of 0.5 × 10^6 ^cells/well to HUVEC monolayers for 60 min. Non-adherent tumor cells were washed off in each sample, the remaining cells were fixed and counted in five different fields (5 × 0.25 mm^2^) using a phase contrast microscope. Mean values were calculated from five counts. Mean adhesion capacity is depicted as adherent cells/mm^2^. One representative of six experiments is shown. * indicates significant difference to controls (p < 0.05).

Single drug application of either 1 μM AEE788 or 1 nM RAD001 induced a slight but significant down-regulation of RCC cell attachment to HUVEC, compared to the untreated controls (fig. 2). Surprisingly, simultaneous exposure of RCC cells to both AEE788 and RAD001 did not always led to a further decrease of the tumor cell attachment rate, compared to the single drug regimen. A stronger response was only seen with respect to KTC-26 but not with respect to the A498 and Caki-1 cells (fig. 2).

**Figure 3:**
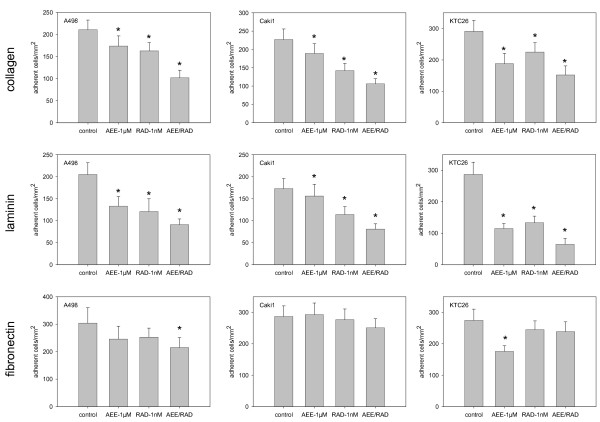
**Adhesion of RCC cells to extracellular matrix proteins**. A498, Caki-1 or KTC-26 cells were treated with 1 μM AEE788 or 1 nM RAD001, applied alone or in combination. Non-treated cells served as the controls. Cells were then added to immobilized collagen, laminin, or fibronectin at a density of 0.5 × 10^6 ^cells/well for 60 min. Plastic dishes were used to evaluate unspecific binding (background control). Non-adherent tumor cells were washed off in each sample, the remaining cells were fixed and counted in five different fields (5 × 0.25 mm^2^) using a phase contrast microscope. Mean values were calculated from the five counts. Specific adhesion capacity (background adhesion on plastic surface was subtracted from adhesion to matrix proteins) is depicted as adherent cells/mm^2^. One representative of six experiments is shown. * indicates significant difference to controls (p < 0.05).

Effects of AEE788 and/or RAD001 on RCC cell binding to extracellular matrix strongly depended on the matrix protein used. RCC cell attachment to collagen was significantly diminished by AEE788 or RAD001, the AEE-RAD combination being more effective than the single drug application (fig. 3). Similarly, interaction of RCC cells with immobilized laminin was blocked distinctly by AEE788 or RAD001, and the combination therapy was superior than the single drug treatment (fig. 3). In contrast, binding of Caki-1 to fibronectin was not influenced neither by the single drug nor by the AEE-RAD combination. KTC-26 binding to fibronectin was blocked by AEE788 exclusively, whereas A498 binding was significantly reduced only when both compounds were used in combination (fig. 3). No drug effects were seen on RCC cell lines grown on Poly-D-Lysin coated dishes.

### AEE788 and RAD001 block RCC cell growth

**Figure 4:**
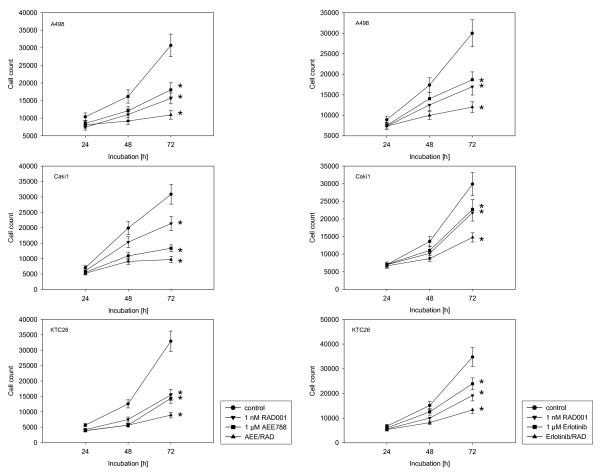
**Effects of RAD001, AEE788 or erlotinib versus drug combination on kidney cancer proliferation in vitro**. A498, Caki-1 or KTC-26 cells were treated with 1 nM RAD001, 1 μM AEE788 or 1 μM erlotinib, applied alone or in combination. Controls remained untreated. Cells were then counted after a further 24, 48 and 72 h using the MTT dye reduction assay. One representative of 6 experiments is shown. * indicates significant difference to controls (p < 0.05).

**Figure 5:**
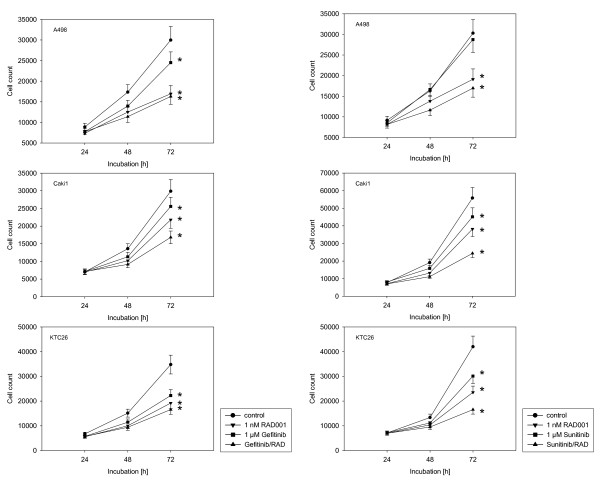
**Effects of RAD001, gefitinib or sunitinib versus drug combination on kidney cancer proliferation in vitro**. A498, Caki-1 or KTC-26 cells were treated with 1 nM RAD001, 1 μM gefitinib or 1 μM sunitinib, applied alone or in combination. Controls remained untreated. Cells were then counted after a further 24, 48 and 72 h using the MTT dye reduction assay. One representative of 6 experiments is shown. * indicates significant difference to controls (p < 0.05).

The proliferative response of RCC to AEE788 and/or RAD001 treatment was analyzed next. Growth of A498, Caki-1 and KTC-26 cells was inhibited significantly by each drug alone. AEE788 and RAD001 induced similar effects on A498 and KTC-26 cells, whereas AEE788 was superior to RAD001 in the Caki-1 cells (fig. 4). The combination of both drugs further decreased the proliferation rate of all RCC cell lines, compared to the single drug application. AEE788 was additionally compared to kinase inhibitors currently in clinical use. The EGF receptor tyrosine kinase inhibitors erlotinib and gefitinib, each applied at 1 μM, significantly reduced RCC cell proliferation (erlotinib > gefitinib; fig. 4+5). However, both agents were not as potent as was 1 μM AEE788. Furthermore, erlotinib-RAD001 and gefitinib-RAD001 combination reduced cell growth to a lesser extent than the AEE788-RAD001 combination. The same was true when the VEGF receptor inhibitor sunitinib was applied (fig. 5). Even, cell growth of A498 was not diminished at all by sunitinib. In all experiments, the Annexin V-FITC assay did not reveal any signs of apoptosis. Therefore, cell growth reduction due to apoptotic events could be excluded. Ongoing studies concentrated on the influence of AEE788 and RAD001 on cell cycle progression and cell cycle regulating proteins.

### AEE788 and RAD001 impair cell cycle progression

**Figure 6:**
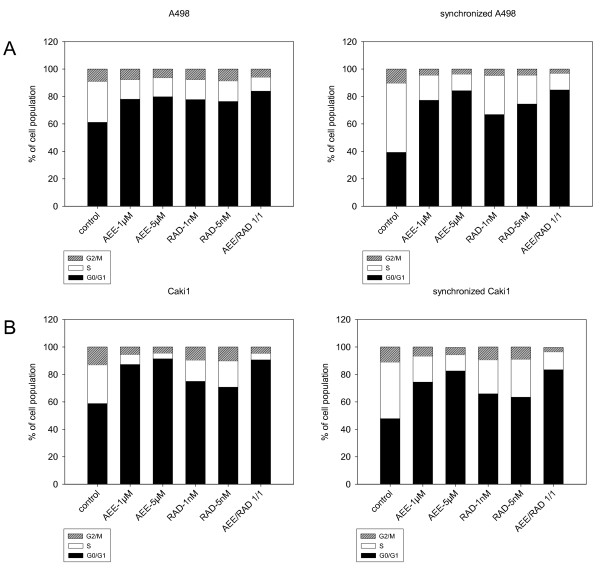
**Cell cycle analysis of A498 (6a) and Caki-1 cells (6b)**. Asynchronous (left) and synchronous (right) cell cultures were used. Cells were treated either with 1 μM or 5 μM AEE788 or with 1 nM or 5 nM RAD001, or with a 1 μM AEE788-1nM RAD001 combination. Controls remained untreated. The cell population at each specific checkpoint is expressed as percentage of the total cells analyzed. One representative experiment of three is shown.

Cell cycle analysis was carried out on A498 and Caki-1 cells. Based on asynchronous A498 cell populations, AEE788 and RAD001 significantly decreased the amount of S-phase and enriched the amount of G0/G1 cells. Both compounds evoked similar effects on A498 cells, independent on the concentration used (1 μM versus 5 μM; fig. 6a, left). Cell cycle progression of asynchronous Caki-1 cells were also affected by AEE788 and RAD001, however AEE788 was more potent than RAD001 in this matter (fig. 6b, left). The maximum cell cycle blockade was achieved when 1 μM AEE788 and 1 nM RAD001 were added to RCC cells in combination.

Subsequently, A498 or Caki-1 cells were released from an aphidicolin block to enrich the mitotic population (fig. 6 a+b, right). In doing so, 1 μM AEE788 or 1 nM RAD001 distinctly delayed cell cycle entry, AEE788 being more effective than RAD001. Combined application of both agents in the 1 μM/1 nM formulation induced much stronger alterations on the cell cycle than the 1 μM AEE788 or 1 nM RAD001 single drug application. Effects induced by the 1 μM/1 nM drug combination were then similar to those seen under 5 μM AEE788 and even more intense than seen under 5 nM RAD001 single drug application.

### AEE788 and RAD001 alter expression level of cell cycle proteins

**Figure 7:**
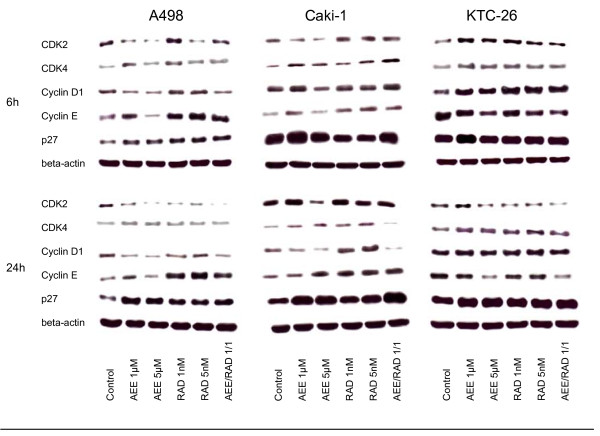
**Western blot analysis of cell cycle proteins, listed in methods**. Asynchronous A498, Caki-1 or KTC-26 cells were treated either with 1 μM or 5 μM AEE788 or with 1 nM or 5 nM RAD001, or with a 1 μM AEE788-1nM RAD001 combination. Controls remained untreated. Drugs were applied for 6 or 24 h. Cell lysates were then subjected to SDS-PAGE and blotted on the membrane incubated with the respective monoclonal antibodies. Beta-actin served as the internal control. The figure shows one representative from three separate experiments.

Alteration of cell cycle regulating proteins strongly depended on the drug exposure time, the drug dosage and the RCC cell line used. With respect to asynchronous A498 cells, cdk2 was lowered after 6 h by 1 or 5 μM AEE788 or by 5 nM RAD001 but enhanced by 1 nM RAD001, compared to the controls (fig. 7). 24 h analysis revealed cdk2 reduction by both AEE788 and RAD001. Cdk4 was found to be up-regulated, notably by 1 μM AEE788 or 1 nM RAD001 after a 6 h exposure. Cyclin D1 was mainly diminished by AEE788 after 6 and 24 h, whereas cyclin E was enhanced after the same time period mainly by RAD001. p27 was drastically elevated after 6 and 24 h by both compounds, compared to the non-treated controls.

AEE788 and RAD001 also manipulated protein expression in asynchronous Caki-1 and KTC-26 cell cultures. Alterations in Caki-1 cells predominantly corresponded to the kind of manipulation in A498 cells (fig. 7). However, major differences were seen in KTC-26 cells, since cdk4 and cyclin D1 became all elevated by AEE788 or RAD001, whereas cyclin E was reduced by AEE788 after a 6 and 24 h drug exposure (fig. 7).

The AEE788-RAD001 combination experiments yielded ambiguous results. Additive effects became obvious in A498 cells with respect to cdk2 expression (24 h), in Caki-1 cells with respect to p27 expression (24 h). This was not true in the KTC-26 cell model. However, cyclin E (6 h, 24 h) was diminished to a greater extent in these cells by the AEE788-RAD001 combination compared to the single drug application.

**Figure 8:**
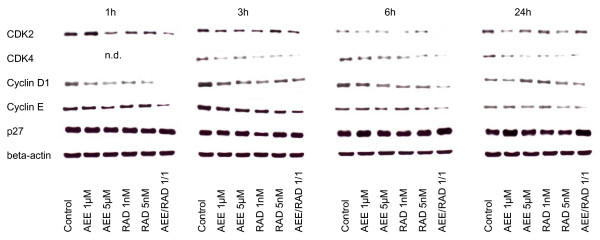
**Western blot analysis of cell cycle proteins, listed in methods**. Synchronized A498 (Fig. 8), Caki-1 (Fig. 9) or KTC-26 cells (Fig. 10) were treated either with 1 μM or 5 μM AEE788 or with 1 nM or 5 nM RAD001, or with a 1 μM AEE788-1nM RAD001 combination. Controls remained untreated. Drugs were applied for 1, 3, 6, or 24 h. Cell lysates were then subjected to SDS-PAGE and blotted on the membrane incubated with the respective monoclonal antibodies. Beta-actin served as the internal control. The figures show one representative from three separate experiments.

**Figure 9:**
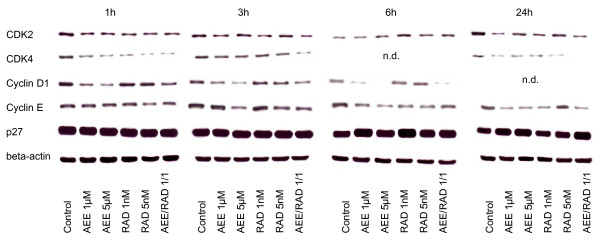
**Western blot analysis of cell cycle proteins, listed in methods**. Synchronized A498 (Fig. 8), Caki-1 (Fig. 9) or KTC-26 cells (Fig. 10) were treated either with 1 μM or 5 μM AEE788 or with 1 nM or 5 nM RAD001, or with a 1 μM AEE788-1nM RAD001 combination. Controls remained untreated. Drugs were applied for 1, 3, 6, or 24 h. Cell lysates were then subjected to SDS-PAGE and blotted on the membrane incubated with the respective monoclonal antibodies. Beta-actin served as the internal control. The figures show one representative from three separate experiments.

**Figure 10:**
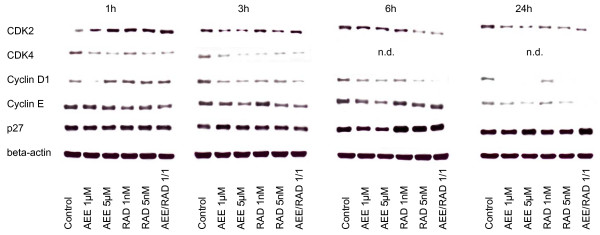
**Western blot analysis of cell cycle proteins, listed in methods**. Synchronized A498 (Fig. 8), Caki-1 (Fig. 9) or KTC-26 cells (Fig. 10) were treated either with 1 μM or 5 μM AEE788 or with 1 nM or 5 nM RAD001, or with a 1 μM AEE788-1nM RAD001 combination. Controls remained untreated. Drugs were applied for 1, 3, 6, or 24 h. Cell lysates were then subjected to SDS-PAGE and blotted on the membrane incubated with the respective monoclonal antibodies. Beta-actin served as the internal control. The figures show one representative from three separate experiments.

When drug treatment and protein analysis was carried out in the synchronous cell culture model, a clearer picture was obtained (fig. 8, 9, 10). As a general rule, cdk2, cdk4, cyclin D1 and cyclin E were all found to be down-regulated by AEE788 or RAD001. Still, few exceptions remained demonstrating no changes or even elevated protein expression, compared to the controls. Alterations of the p27 expression level took place 6 and 24 h after the experimental start, becoming enhanced in A498 and Caki-1 cells by AEE788. The same effect was evoked by RAD001 in Caki-1. Interestingly, AEE788 reduced p27 expression in KTC-26 cells, whereas RAD001 enhanced it (6 h analysis).

AEE788-RAD001 combination treatment strongly augmented the effects of the single drug treatment in all cell lines investigated. In particular, cdk2, cdk4, cyclin D1 and cyclin E were drastically reduced or even lost at specific time points in A498 and KTC-26 cells when both agents were used together.

### Analysis of mTOR and EGF receptor signaling

**Figure 11:**
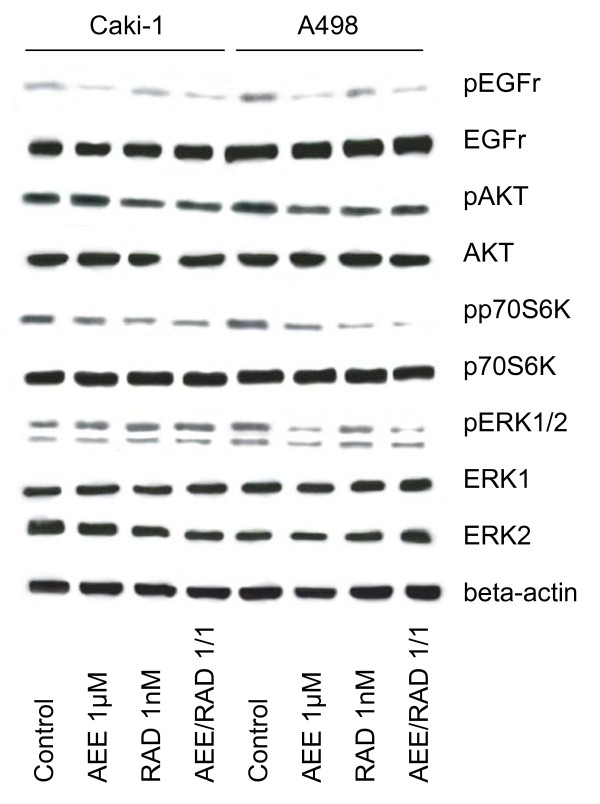
**Western blot analysis of cell signaling proteins, listed in methods**. A498 or Caki-1 cells were treated either with 1 μM AEE788 or with 1 nM RAD001, or with a 1 μM AEE788-1nM RAD001 combination. Controls remained untreated. Drugs were applied for 24 h. Cells were then kept for 2 h in serum-free cell culture medium and subsequently stimulated for 30 min with EGF (100 ng/ml). Cell lysates were subjected to SDS-PAGE and blotted on the membrane incubated with the respective monoclonal antibodies. Beta-actin served as the internal control. The figure shows one representative from three separate experiments.

Finally, we evaluated if AEE788 and/or RAD001 effects are linked to the inhibition of their primary targets. Total EGF receptor, ERK1/2, Akt and p70S6K were not changed by both agents (fig. 11). However, amount of activated EGF receptor was diminished by AEE788 in Caki-1 and A498 cells. Activated EGF receptor was also found to be reduced in presence of the AEE788-RAD001 drug combination. Phosphorylated ERK1/2 became lost by AEE788 or the AEE788-RAD001 drug combination in A498 cells. This phenomenon was not seen in Caki-1 cells. Interestingly, activation of Akt was only slightly down-regulated by RAD001 in A498 cells, and the response of Caki-1 cells to RAD001 was only marginal in this matter. However, RAD001 strongly inhibited p70S6K activation in both Caki-1 and A498 cells. Very strong deactivation of p70S6K was achieved by the AEE788-RAD001 drug combination in A498 cells.

## Discussion

AEE788 is a 7H-pyrrolo [2,3-*d*]pyrimidine-class receptor tyrosine kinase inhibitor that potently inhibits the EGFR associated kinase activity (IC_50_: 2 nM) with additional inhibition of VEGFR-1 (Flt-1; IC_50_: 59 nM) and VEGFR-2 (kinase domain region/Flk-1; IC_50_: 77 nM) at higher concentrations [12]. Anti-proliferative effects of this compound have already been demonstrated on prostate [13], colon [14], pancreatic [15], lung, ovarian [16], and glioblastoma cell lines [17]. Evidence is presented here showing that AEE788 in the μM range interferes with the RCC-endothelium and RCC-matrix communication and alters RCC cell growth dynamics.

A significant decrease of S-phase and concomitant increase of G0/G1 phase cells was seen in the presence of AEE788 accompanied by distinct modifications of cell cycle regulating proteins. The data were more concise in the synchronous than in the asynchronous cell culture model, which is not surprising because specific effects of AEE788 on mitotic events may become more obvious in a homogeneous cell population. Indeed, Peng and coworkers reported that the activity of a particular drug limited to certain cell cycle phases may be diluted under asynchronous conditions [18]. Based on the synchronous cell culture model, cdk2, cdk4, cyclin D1 and cyclin E were all found to be reduced, whereas p27 was up-regulated by AEE788 in the RCC cell lines.

These findings are important since disturbances of cell cycle control in the tumorigenesis of RCC have recently been shown to be paralleled by elevation of cyclin D1 and cdk4, accompanied by the attenuation of p27 expression [19]. Inline with the *in vitro *data, analysis of tumor specimen taken from RCC patients revealed a correlation between cyclin D1 and cyclin E protein level and the tumor proliferation index [20]. Vice versa, an inverse correlation was seen between p27 expression and tumor size, and RCC patients with p27 low tumors had poorer survival than patients with p27 high tumors [21,22].

Obviously, cyclin D1, cyclin E, cdk4 and p27 represent pivotal elements in RCC cells and targeting these proteins may become an intriguing option to stop RCC progression. In fact, incubation of RCC cells with thiazolidinedione decreased the protein levels of cyclin D1 and cdk4, and increased the levels of p27 which altogether led to G0/G1 arrest and massive tumor cell apoptosis [23]. A similar phenomenon has been observed by others treating RCC cells with the short chain fatty acid sodium butyrate or phenylacetate [24,25].

The data presented here point to a powerful anti-tumoral activity of AEE788. Nevertheless, AEE788 did not reduce cyclin D1, cyclin E, cdk2 and cdk4 at all time points analyzed. Cdk1 became even (slightly) enhanced in synchronized KTC-26 and A498 cells after 1 h (1 μM application). Therefore, it may be assumed that AEE788 does not completely suppress cell mitosis but rather slows down the mitotic cycle. In line with this speculation, the proliferative activity of RCC cells presented in figure 4 was drastically down-regulated, though not totally blocked by AEE788.

RAD001, the 40-O-(2-hydroxyethyl) derivative of rapamycin [26], blocks proliferation of several tumor cell lines *in vitro*. No detailed analysis has been carried out on RCC cell lines. However, clinical trials confirm the relevance of targeting the mTOR pathway in RCC [27-29]. RAD001 has recently been shown to exhibit a partial response (23%) and stable disease (38%) in a phase II trial of patients with RCC. Progression-free survival was 11.2 months [30]. Another phase II trial evaluating RAD001 was presented at ASCO 2008 and shows encouraging anti-tumor activity in RCC patients which have had prior exposure to sorafenib or sunitinib [31]. Finally, treatment with RAD001 prolonged progression-free survival relative to placebo in patients with metastatic RCC in a phase III study [32].

We present evidence that RAD001 significantly influences RCC adhesion and growth behaviour. RAD001 had a distinct impact on the suppression of cellular S-phase fraction and modification of cell cycle protein expression. Remarkably, RAD001's effects on cell cycle proteins did not always parallel the characteristics of AEE788. Notably, cyclin D1 were found to be reduced by AEE788 in synchronized Caki-1 cells but remained unchanged in the presence of RAD001 at a particular time point. It is not clear if cyclin D1 is incompletely targeted by RAD001 (particularly in Caki-1 cells) or if RAD001 acts in a different manner than AEE788. Studies on malignant glioblastoma cells revealed both compounds to affect cellular proliferation in different ways [33]. Therefore, non-overlapping mechanisms should be considered when interpreting our data. This is an important issue, as some targeted therapies require the cell to enter specific cell cycle points to induce therapeutic effects.

As the most important message, simultaneous use of both AEE788 and RAD001 offered a distinct combinatorial benefit and thus may provide a therapeutic advantage over either agent as monotherapy for RCC treatment. This is highly relevant, since single agents rarely induced complete responses in clinical trials, presumably due to compensatory cross-talk among receptors within a signaling network as well as with heterologous receptor systems in RCC cells. Combinations of targeted agents could improve limited therapeutic efficacy and overcome resistance that might develop under single-agent therapy. At the present, two different concepts of combination targeted therapy for RCC are discussed. "Horizontal blockade" is aimed to concurrently target numerous molecules involved in RCC proliferation and dissemination (e. g. simultaneous targeting of the EGF and VEGF receptor). The other popular concept of "vertical blockade" is aimed to target the same pathway at two or more different levels.

Concerning the latter, synergistic effects were seen in several tumor cell lines when both mTOR and EGF receptor inhibitors were administrated in combination [34-36]. Recent data suggest that combining mTOR with VEGF receptor inhibitors may have clinical potential to enhance survival of cancer patients [37,38].

The present study was designed to interfere with the tumor cell signaling network horizontally and vertically by targeting the VEGF receptor and EGF receptor as well as the mTOR-Akt axis. The combinatorial impact of AEE788 and RAD001 was mainly seen in the suppression of RCC proliferation. Results of the adhesion experiments are not clear. Additive effects became evident with respect to KTC-26 adhesion but not with respect to A498 and Caki-1 adhesion to HUVEC. AEE788-RAD001 combination treatment also blocked RCC cell binding to laminin and collagen to a higher extent than the monotherapy did. However, this was not true in the fibronectin assay. Based on our *in vitro *model, we postulate that synergism may not be evoked against all the events in the evolution of neoplastic disease and metastatic tumor dissemination. Presumably, combinatorial application of AEE788 and RAD001 may be favourable in blocking tumor growth, whereas therapeutic modulation of tumor transmigration may be limited to specific phases of the tumor cell invasion cascade. Nevertheless, no data are available dealing with this issue and, therefore, this is still speculative. Further experiments are necessary to demonstrate how the drugs modify RCC adhesion and migration behaviour, and to characterize the relevant target proteins.

## Conclusion

Our results indicate that the receptor tyrosine kinase inhibitor AEE788 and the mTOR inhibitor RAD001 both act on RCC cell adhesion and cell growth. Combined use of both compounds seems to be more effective than single drug application. This view is supported by findings in glioblastoma cell lines, where the combination of AEE788 and RAD001 resulted in increased rates of cell cycle arrest and apoptosis and reduced proliferation more than either agent alone [33]. Therefore, simultaneous use of both AEE788 and RAD001 may offer a distinct combinatorial benefit and thus may provide a therapeutic advantage over either agent as monotherapy for RCC treatment. Animal experiments are necessary to deepen the in vitro findings. Since VEGF receptors are strongly involved in angiogenic events, the anti-angiogenic potential of both drugs should also be evaluated in the in vivo model.

## Competing interests

The authors declare that they have no competing interests.

## Authors' contributions

EJ performed all major experimental work of the study. JE and AM carried out western blotting and cell cycle analysis. IN performed the adhesion and binding studies. LH established the cell synchronization protocol. RAB contributed to the design and coordination of the study and drafted the manuscript. DJ participated in the conception and design of the study. JJ was involved in the overall design of the study and data interpretation, and helped to draft the manuscript.

## Pre-publication history

The pre-publication history for this paper can be accessed here:

http://www.biomedcentral.com/1471-2407/9/161/prepub
